# Investigation of Biopsied Non-Plaque-Induced Gingival Lesions in a Turkish Population: A 5-Year Retrospective Study

**DOI:** 10.5152/eurasianjmed.2023.0088

**Published:** 2023-06-01

**Authors:** Alparslan Dilsiz, Sema Nur Sevinç Gül

**Affiliations:** 1 Department of Periodontology, Atatürk University, Faculty of Dentistry, Erzurum, Turkey

**Keywords:** Non-plaque-induced gingival lesions, reactive gingival lesions, gingival premalignant neoplasms, gingival malignant neoplasms

## Abstract

**Objective:**

The study aimed to analyze the distribution and frequency of individuals diagnosed with histopathologically non-plaque-induced gingival lesions and categorize them according to the non-plaque-induced gingival disease classification published at the 2017 World Workshop of Periodontology.

**Materials and Methods:**

Clinical features of the gingival lesion with histopathological diagnosis data in the period 1998-2003 were retrospectively analyzed . The lesions were classified as reactive lesions, malignant neoplasms, premalignant neoplasms, autoimmune disorders, benign neoplasms, hypersensitive reactions, and genetic lesions. Their distribution according to age, gender, histopathological diagnosis, and oral sites was examined. Variables were analyzed using descriptive statistics.

**Results:**

Among a total of 217 biopsied gingival samples, the most frequent pathologic nature of biopsied non-plaque gingival lesions were reactive lesions (n = 80, 36.87%) and premalignant neoplasms (n = 64, 29.49%). In addition, the 5 most frequent types of all cases included pyogenic granuloma (n = 45, 20.74%), epithelial dysplasia (n = 40, 18.43%), papilloma (n = 33, 15.21%), epithelial hyperplasia (n = 24, 11.06%), and calcifying fibroblastic granuloma (n = 13, 5.99%).

**Conclusions:**

In a Turkish population, the most frequently biopsied non-plaque-induced gingival lesions were reactive lesions and premalignant neoplasms. This study shows that the types of lesions that clinicians, in general, especially periodontologists, can expect to encounter in their practice are the most frequently applied gingival lesions.

**Main Points**

- This study provides information about gingival pathologies. This article may give an idea for the histopathological diagnosis of gingival pathologies by the general dentist.
- The most frequently biopsied non-plaque gingival lesions were reactive lesions and premalignant neoplasms in a Turkish population.
- Dentists and medical practitioners should be careful about gingival lesions and precancerous and malignant pathologies.

## Introduction

The oral cavity is a complex structure in the head and neck region. It consists of various structures such as jaws, teeth, tongue, salivary glands, and soft and hard palate.^1^ As the oral mucosa is always under the effect of various internal and external stimuli, it occurs in various developmental disorders, irritation, inflammation, and neoplastic lesions.^2^

Reactive lesions are hyperplastic structures caused by chewing the oral mucosa, poor oral hygiene, fractured teeth, and extended denture flanges.^3^ Pyogenic granuloma, fibrous epulis, peripheral giant cell granuloma, and calcifying fibroblastic granuloma are the most common reactive lesions of the oral cavity.^4^

Neoplastic oral mucosal lesions are divided into premalignant lesions and malignant lesions. Premalignant lesions are examined under the heading of leukoplakia and erythroplakia, and malignant lesions are classified as squamous cell carcinoma, leukemia, and lymphoma.^5^

It is very important to know both the clinical features and the pathology of the lesions for the recognition, diagnosis, and treatment of common oral diseases. When any oral lesion is detected, the symptom, size, location, color, elapsed time, and the biopsy of the lesion should be taken and histopathologically evaluated. According to the European Federation of Periodontology (EFP) and the American Academy of Periodontology (AAP) 2017 World Workshop, non-plaque-induced gingival diseases are classified as genetic lesions, specific infections, hypersensitive reactions, autoimmune diseases of the skin and mucous membranes, granulomatous inflammatory conditions, reactive processes, premalignant neoplasms, malignant neoplasms, endocrine, nutritional and metabolic diseases, traumatic lesions, and gingival pigmentation.

This retrospective study aims to determine the frequency and distribution of oral lesions obtained from patients of all age and gender groups who applied to the Faculty of Dentistry, Atatürk University, diagnosed histopathologically, categorized according to the non-plaque-induced gingival disease classification published at the 2017 World Workshop and bring them to the literature.

## Materials and Methods

A retrospective study was performed on biopsied gingival lesions collected over 5 years in the Atatürk University, Faculty of Dentistry. This study was independently reviewed and approved by The Institutional Internal Review and Ethics Board (April 19, 2009 No: 017) and conducted according to the 2008 Declaration of Helsinki and later amendments.

Medical records of all patients who underwent a biopsy during this period were evaluated. Patients with a lesion in the gingiva with defined histopathological diagnosis and patients with sufficient demographic information were included in the study. Patients with the following disorders were excluded from the study: (1) disorder of salivary gland, (2) bone lesions extending to the gingiva, and (3) plaque-induced-gingival diseases. An informed consent form was obtained from the patients included in the study. According to their histopathological diagnoses, pathologies belonging to 217 cases, 116 females and 101 males, the lesions are classified as reactive lesions, malignant neoplasms, premalignant neoplasms, autoimmune disorders, benign neoplasms, hypersensitive reactions, and genetic lesions. In the tables of the groups, the number of cases, average age, gender, sex ratio, frequency of lesions in their group, and incidence rates according to all lesions are given.

Chi-square test and descriptive statistics were applied to the data obtained, and their distribution according to age and gender was examined. All analyses were performed using Statistical Package for the Social Sciences® software version 20 (IBM Corp., Armonk, NY, USA). Among the results obtained, those with *P* < .05 were considered significant.

## Results

The most frequent histopathologic groups, along with age, sex, and location distributions, are summarized in Table 1.

According to Table 1, the 5 most frequent types of all cases included pyogenic granuloma (n = 45, 20.74%), epithelial dysplasia (n = 40, 18.43%), papilloma (n = 33, 15.21%), epithelial hyperplasia (n = 24, 11.06%), and calcifying fibroblastic granuloma (n = 13, 5.99%). The sixth most common diagnoses are peripheral giant cell granuloma (n = 12, 5.53%) and lichen planus (n = 12, 5.53%). Hereditary gingival fibromatosis was the seventh most frequent type (n = 11, 5.06%). In addition, one of the hyperplastic lesions, fibrous epulis, was the eighth diagnosis (n = 10, 4.61%). The ninth most frequent diagnosis and most common type of cancer was squamous cell carcinoma (n = 8, 3.69%).

Gingival lesions were diagnosed in patients with a wide range of ages, from 6 to 80 years, with a mean age of 37.49 years.

Table 1 also shows a comparison of gender and location; 53.5% (n = 116) of total cases were females, and 46.5% (n=101) were males.

In addition, a slight majority of cases were obtained from maxillary gingiva (n = 122, 56.2%), more than mandibular gingiva (n = 95, 43.8%).

To classify these different types of lesions into more specific categories, we divided the lesion types into several groups according to their pathological characteristics (Table 2).

As shown in Table 2, the most frequently observed biopsied lesions were “reactive lesions” (n = 80), reaching up to 36.87% of all lesions. In “reactive lesions,” the largest proportion was pyogenic granuloma, followed by calcifying fibroblastic granuloma, peripheral giant cell granuloma, and fibrous epulis.

## Discussion

In this study, we conducted a retrospective study to investigate the frequency and distribution of the biopsied non-plaque-induced gingival lesions analyzed in a Turkish population. We arranged and identified the current results of the new classification of gingival health and gingival diseases/conditions established in the 2017 World Workshop by EFP and AAP. The existing gingival lesions were divided into 7 groups. The most common and least seen are reactive lesions, premalignant neoplasms, benign neoplasms, autoimmune disorders, genetic lesions, malignant neoplasms, and hypersensitive reactions.

The diagnostic classifications of biopsies taken in previous studies were categorized into 3 types: non-neoplastic lesions, benign lesions, and malignant lesions,^6-8^ and the majority of biopsied samples were non-neoplastic lesions. Consistent with the previous reports,^6-9^ most of the lesions we examined were reactive lesions, with 36.87% of all lesions. Among these, pyogenic granuloma is the most common diagnosis, with a rate of 20.74%. The results of this study are consistent with previous studies.^7,10,11^ About 55.6% of cases of pyogenic granuloma were found in maxillary gingiva, which was lower than those reported by Ababneh^11^ (64%) and Alblowi^12^ (57.73%) and higher than those reported by Zhang et al^13^ (47.10%). Pyogenic granuloma represented nearly one-fifth of all gingival lesions, with a peak incidence of appearance at 36.7 years, which was somewhat older than previous reports. It is more common in females in our study population, which is inconsistent with earlier reports.^6,7,10-12,14^ Pyogenic granuloma was followed by peripheral giant cell granuloma, calcifying fibroblastic granuloma, and fibrous epulis.

Malignant neoplasms of lesions were calculated as 3.69%. Squamous cell carcinoma (SCC) was the only one malignant lesion reported in gingival biopsy specimens in this study, which was consistent with the outcomes of other reports.^6,8,10^ In our study, SSC most frequently appeared in the sixth decade, and only 1 case was detected below 40 years. We also detected that the mean age of SCC was 60.13, fewer than that of Makridis et al.^15^

In this study, premalignant neoplasms resulted in about 30% of all biopsies; oral premalignant neoplasms include oral leukoplakia, oral lichen planus, lichenoid lesions, and oral erythroplakia. Oral leukoplakia is categorized according to the presence or absence of epithelial dysplasia, classified as without epithelial dysplasia and with epithelial dysplasia; 18.43% of the investigated group were diagnosed with epithelial dysplasia of different degrees. Li et al^9^ reported a prevalence of gingival epithelial dysplasia of 6.95%. Previous studies described the prevalence of leukoplakia without dysplasia ranging between 18% and 38%,^16-18^ while our study has detected a prevalence of gingival without epithelial dysplasia of 11.06% within the studied population. Other studies have reported the frequency of leukoplakia without epithelial dysplasia as 6.36% and 4.94%, respectively.^9,19^ The difference in these results may be related to the population studied. Our study determined that these lesions were generally seen in the mandible, and these data are compatible with the previous study.^9^ However, these lesions were detected more frequently in the maxilla region in other studies.^6,19^ This difference may be due to the differences in life expectancy between the regions where the studies were conducted, as leukoplakia is affected by more than 1 etiological factor. For example, tobacco and alcohol consumption are the most important etiological factors. However, other etiological factors include human papillomavirus (HPV), tooth restoration, mechanical irritation, candidiasis, low serum vitamin A, and carotene.

The autoimmune disorders were investigated under the title of lichen planus and pemphigus vulgaris. The prevalence of lichen planus was determined as 5.53%, which was slightly higher than reported by Alblowi et al^12^ (5%) and Li et al^9^ (4.23%). The frequency of the other study diagnosed with lichen planus on biopsy specimens was quite low, which was inconsistent with our study.^19^ On the other hand, Carbone et al^6^ reported that the prevalence of oral lichen planus is just about 10%.^6^ Pemphigus vulgaris was detected in only 2 cases (0.92%), they were on average 50.5 years old, and both were male. While the incidence of pemphigus vulgaris was 2.41% in one study,^6^ it was 1.25% in another study.^9^ We think these differences may be related to the sample size of the study group and the patient population.

Benign neoplasms were usually detected in the gingiva; papilloma is the most prevalent (15.21%). It is known that squamous cell papilloma, thought to be caused by HPV, is transmitted from mother to child and spouses to each other. Our results are inconsistent with previous studies due to its infectious pathology.^6,9,19,20^ Hemangioma, another benign neoplasm, was detected at a rate of 1.84%, and this rate is slightly higher than other studies.^6,19^

Plasma cell gingivitis, which is one of the hypersensitive reactions, is a rare condition characterized by a hypersensitivity response in the gingival tissue. Plasma cell gingivitis was detected in only 3 patients (1.38%). Although the cases were mostly seen in women, we observed it in 2 males and 1 female. The mean age is 36.33, which was more common in the maxilla.

Hereditary gingival fibromatosis is a genetic disease characterized by gingival enlargement and is a frequently encountered phenomenon. Hereditary gingival fibromatosis was encountered in approximately 5% of our study group, which is considerably higher than in previous studies.^9,19^ Since this genetically inherited disease can be seen in all family members, the high rate can be explained for this reason. The prevalence of consanguineous marriages in this area was thought to be one of the factors that increased the frequency.

One of the limitations of this study is that the number of samples is limited due to collection from only 1 center. The other limitation is some inconsistencies when categorizing pathologically diagnosed biopsy lesions by pathological nature classification and new gingival disease classification. The benign neoplasm option was not available in the new classification; hemangioma and papilloma were collected under the title of benign neoplasms in this study. Papilloma has been evaluated in the new classification of infectious diseases of viral origin. However, the benign neoplasm title was preferred because there was no other case of infectious origin in our study, and there was no title in the 2018 classification for hemangioma.

As in all pathological formations, the relationship between the primary lesion and subsequent histological findings in non-plaque gingival pathologies is important for formulating a diagnostic hypothesis with specific clinical features. In the study of the American Board of Specialists in Oral Medicine examining the current approaches for the diagnosis and treatment of oral premalignant lesions, it was reported that most of the clinicians refer to the initial clinical diagnosis before taking a biopsy to make a diagnosis.^21^ While this may be useful for starting treatment without delay, for this approach to be successful, the initial clinical diagnosis must be correct and no features have been overlooked. For this reason, it has become crucial to investigate the level of accuracy of clinical diagnoses made by clinicians against the definitive diagnosis reached by histopathological examination.^22^

In conclusion, the most frequently biopsied non-plaque gingival lesions were reactive lesions and premalignant neoplasms in a Turkish population. The types of lesions that clinicians in general and periodontists, in particular, can expect to encounter in their practice, as this study shows that they are the most frequently consulted gingival lesions. Considering the relationship between clinical diagnosis and histological findings, the accuracy of the initial clinical diagnosis can be increased by developing diagnostic hypothesis formulations.

## Figures and Tables

**Table 1.** Frequencies of Various Histopathologic Groups with Age, Gender, and Location Distributions

Classification	N	Percentage (%)	Age (years) Mean ± SD	Gender		Location	
				Female N (%)	Male N (%)	Mand N (%)	Max N (%)
Pyogenic granuloma	45	20.74	36.73 **± **20.39	29 (64.4)	16 (35.6)	20 (44.4)	25 (55.6)
Epithelial dysplasia	40	18.43	35.2 **± **17.31	22 (55.0)	18 (45.0)	14 (35.0)	26 (65.0)
Papilloma	33	15.21	40.30 **± **21.25	16 (48.5)	17 (51.5)	13 (39.4)	20 (60.6)
Epithelial hyperplasia	24	11.06	35.29 **± **17.03	9 (37.5)	15 (62.5)	9 (37.5)	15 (62.5)
Calcifying fibroblastic granuloma	13	5.99	21.92 **± **13.18	8 (61.5)	5 (38.5)	7 (53.8)	6 (46.2)
Peripheral giant cell granuloma	12	5.53	27.42 **± **15.76	8 (66.7)	4 (33.3)	10 (83.3)	2 (16.7)
Lichen planus	12	5.53	31.75 **± **13.22	9 (75.0)	3 (25.0)	4 (33.3)	8 (66.7)
Hereditary gingival fibromatosis	11	5.06	30.27 **± **19.87	4 (36.4)	7 (63.6)	3 (27.3)	8 (72.7)
Fibrous epulis	10	4.61	63.8 **± **10.67	6 (60.0)	4 (40.0)	8 (80.0)	2 (20.0)
Squamous cell carcinoma	8	3.69	60.13 **± **14.21	2 (25.0)	6 (75.0)	4 (50.0)	4 (50.0)
Hemangioma	4	1.84	60.0 **± **17.15	2 (50.0)	2 (50.0)	2 (50.0)	2 (50.0)
Plasma cell gingivitis	3	1.38	36.33 **± **22.14	1 (33.3)	2 (66.7)	1 (33.3)	2 (66.7)
Pemphigus vulgaris	2	0.92	50.5 **± **10.61	0	2 (100)	0	2 (100)
Total	**217**	**100.0**	**37.49**	**116 (53.5)**	**101 (46.5)**	**95 (43.8)**	**122 (56.2)**

SD, standard deviation.

**Table 2.** The Number and Frequency of Different Biopsied Lesions According to Pathological Nature

Pathologic Nature	Total, n (%)	Histopathological Diagnosis
Reactive lesions	80 (36.87)	Pyogenic granuloma Peripheral giant cell granuloma Calcifying fibroblastic granuloma Fibrous epulis
Premalignant neoplasms	64 (29.49)	Epithelial dysplasia Epithelial hyperplasia
Benign neoplasms	37 (17.05)	Papilloma Hemangioma
Autoimmune disorders	14 (6.45)	Pemphigus vulgaris Lichen planus
Genetic lesions	11 (5.07)	Hereditary gingival fibromatosis
Malignant neoplasms	8 (3.69)	Squamous cell carcinoma
Hypersensitive reactions	3 (1.38)	Plasma cell gingivitis
Total	**217 (100)**	
